# 2135. Epetraborole *in vitro* Activity Against *Mycobacterium avium* complex Recent Clinical Isolates from Japan

**DOI:** 10.1093/ofid/ofad500.1758

**Published:** 2023-11-27

**Authors:** Tiffany Keepers White, Akio Aono, Yuriko Igarashi, Kinuyo Chikamatsu, Akiko Takaki, Satoshi Mitarai

**Affiliations:** AN2 Therapeutics, Menlo Park, California; The Research Institute of Tuberculosis Japan Anti-Tuberculosis Association, Kiyose, Tokyo, Japan; Research Institute of Tuberculosis, kiyose, Tokyo, Japan; The Research Institute of Tuberculosis, Japan Anti-Tuberculosis Association, Kiyose, Tokyo, Japan; Research Institute of Tuberculosis, Japan Anti-Tuberculosis Association, Kiyose, Tokyo, Japan; Research Institute of Tuberculosis, kiyose, Tokyo, Japan

## Abstract

**Background:**

Epetraborole (EBO) is a boron-containing, oral inhibitor of bacterial leucyl-tRNA synthetase, an essential enzyme in protein synthesis. EBO demonstrates potent activity against nontuberculous mycobacteria and is currently under clinical development for treatment of treatment-refractory *Mycobacterium avium* complex (MAC) lung disease. The objective of this study was to evaluate the in vitro activity of EBO against recent MAC isolates from Japan.

**Methods:**

Minimal inhibitory concentration (MIC) values for EBO, amikacin (AMK), clarithromycin (CLR), rifabutin (RFB), and ethambutol (EMB) were determined using broth microdilution assays according to Clinical and Laboratory Standards Institute M24-A3 guideline (2018) against 110 MAC clinical isolates collected from Japanese patients in 2020. MAC isolates were of respiratory origin and included 55 *M. avium* and 55 *M. intracellulare* isolates.

**Results:**

EBO (MIC) values ranged from 0.25 - 16 µg/mL and the EBO MIC_50_ and MIC_90_ were 2 and 4 µg/mL, respectively (Table 1). The CLR MIC range was 0.125 - > 32 µg/mL and included 4 CLR-resistant isolates (MIC ≥ 32 µg/mL). CLR MIC_50_ and MIC_90_ were 1 and 4 µg/mL, respectively. All isolates were susceptible to AMK and had an MIC range of 2 - 32 µg/mL; the MIC_50_ and MIC_90_ were 8 and 16 µg/mL, respectively. The EMB MIC range was 2 - > 32 µg/mL; the MIC_50_ and MIC_90_ were 4 and 16 µg/mL, and the RFB MIC range was ≤0.03 - 2 µg/mL and MIC_50_ and MIC_90_ were 0.06 and 0.25 µg/mL. EBO maintained activity with MIC values of 0.25 - 2 µg/mL against the 4 CLR-resistant isolates.

Epetraborole and comparator antimicrobial minimum inhibitory concentration values against 110 recent Mycobacterium avium complex clinical isolates from Japan

**Figure:**
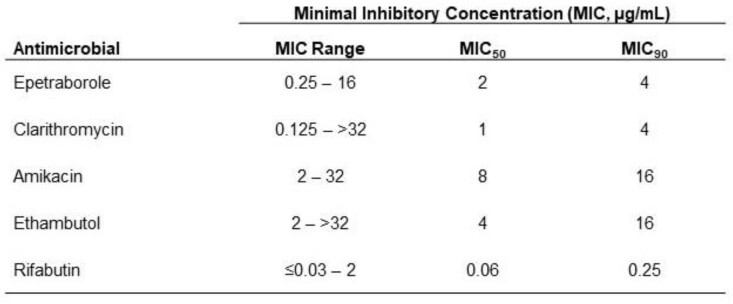

MIC=minimal inhibitory concentration; MIC50=minimal inhibitory concentration for 50% of isolates tested; MIC90=minimal inhibitory concentration for 90% of isolates tested.

**Conclusion:**

EBO demonstrated potent in vitro activity against 110 recent MAC isolates collected from Japanese patients. Furthermore, EBO demonstrated in vitro activity against CLR-resistant MAC isolates suggesting that CLR-resistance does not impact EBO activity. These data are similar to results obtained against U.S. isolates and support the continued clinical evaluation of the use of EBO in the treatment of MAC lung disease in Japan.

**Disclosures:**

**Tiffany Keepers White, PhD**, AN2 Therapeutics: Employee|AN2 Therapeutics: Stocks/Bonds **Satoshi Mitarai, MD, PhD**, AN2 Therapeutics, Inc.: Grant/Research Support

